# Challenges in implementing GP clusters in Scotland: a qualitative study comparing the views of senior primary care stakeholders in 2016 with those in 2021

**DOI:** 10.3399/BJGPO.2022.0152

**Published:** 2023-05-17

**Authors:** Catherine Kidd, Eddie Donaghy, Huayi Huang, Rhian Noble-Jones, Sharon Ogilvie, Julia McGregor, Margaret Maxwell, John Gillies, David AG Henderson, Harry HX Wang, Stewart W Mercer

**Affiliations:** 1 College of Medicine and Veterinary Medicine, University of Edinburgh, Edinburgh, UK; 2 Usher Institute, College of Medicine and Veterinary Medicine, University of Edinburgh, Edinburgh, UK; 3 Nursing and Health Care, School of Medicine, University of Glasgow, Glasgow, UK; 4 Nursing, Midwifery, and Allied Health Professionals Research Unit, University of Stirling, Scotland, UK; 5 School of Public Health, Sun Yat-Sen University, Guangzhou, China

**Keywords:** primary care reform, clusters, general practice, quality of health care, qualitative research

## Abstract

**Background:**

Formation of GP clusters began in Scotland in April 2016 as part of a new Scottish GP contract. They aim to improve the care quality for local populations (intrinsic role) and the integration of health and social care (extrinsic role).

**Aim:**

To compare predicted challenges of cluster implementation in 2016 with reported challenges in 2021.

**Design & setting:**

Qualitative study of senior national stakeholders in primary care in Scotland.

**Method:**

Qualitative analysis of semi-structured interviews with 12 senior primary care national stakeholders in 2016 (*n* = 6) and 2021 (*n* = 6).

**Results:**

Predicted challenges in 2016 included balancing intrinsic and extrinsic roles, providing sufficient support, maintaining motivation and direction, and avoiding variation between clusters. Progress of clusters in 2021 was perceived as suboptimal and was reported to vary significantly across the country, reflecting differences in local infrastructure. Practical facilitation (data, administrative support, training, project improvement support, and funded time) and strategic guidance from the Scottish Government was felt to be lacking. GP engagement with clusters was felt to be hindered by the significant time and workforce pressures facing primary care. These barriers were considered as collectively contributing to cluster lead ‘burnout’ and loss of momentum, exacerbated by inadequate opportunities for shared learning between clusters across Scotland. Such barriers preceded, but were perpetuated by, the impact of the COVID-19 pandemic.

**Conclusion:**

Apart from the COVID-19 pandemic, many of the challenges reported by stakeholders in 2021 were predicted in 2016. Accelerating progress in cluster working will require renewed investment and support applied consistently across the country.

## How this fits in

GP cluster working was introduced nationally by the Scottish Government following the abolition of the Quality and Outcomes Framework (QOF) in April 2016, formalised in a new Scottish GP contract in April 2018. Clusters aim to improve the quality of care for local populations (intrinsic role) and facilitate the integration of health and social care (extrinsic role), but there is little information on the progress of clusters compared with expectations and predicted challenges when they were first formed. This qualitative study, based on interviews with national primary care stakeholders in 2016 and 2021, indicates that many of the challenges reported in the more recent interviews were predicted, including tensions between the intrinsic and extrinsic roles of clusters, the need for support to maintain motivation and direction, and variation in progress geographically. In 2021, progress was perceived as suboptimal owing to these barriers that preceded, but were perpetuated by, the impact of the COVID-19 pandemic. Accelerating progress in cluster working will require renewed investment and support applied consistently across the country.

## Introduction

GP clusters were introduced in Scotland in April 2016, replacing the QOF pay-for-performance programme that had dominated the landscape of primary care quality improvement across the UK since 2004.^1^ This change represented a significant shift from the externally driven, incentive-based methodology embodied by the QOF, towards one centred around more holistically meeting the needs of local populations (see Supplementary file 1 for further details).^2,3^ Cluster working, together with expansion of the multidisciplinary team (MDT), became formalised in the new Scottish GP contract in April 2018.^4^

Clusters are geographical groupings of 5–8 GP practices, each represented by a nominated practice quality lead (PQL), who works collaboratively to engage in peer-led quality improvement activity relevant to their local population.^2–4^ Each cluster has an identified GP cluster quality lead (CQL), responsible for providing a leadership role in coordinating quality improvement activities both within, and on behalf of, their GP cluster, and for liaison with relevant local and professional organisations.^2,3^

The aspirations for cluster working are set out in the ‘Improving Together’ framework,^2^ which subdivides their intended functions into intrinsic (improving quality of care across practices within the cluster) and extrinsic (engaging in the broader integration of health and social care locally) roles. While the high-level expectations for GP clusters have been clearly defined, the progress of clusters towards achieving these intended impacts over the past 6 years is less clear. The limited available literature suggests that GP clusters may not have progressed at a pace that was envisaged at the time of their conception.^5–7^

As such, this longitudinal qualitative study aimed to explore and compare the expected progress of GP clusters, as reported by key senior national stakeholders in primary care in Scotland (from interviews conducted in 2016) with more recent views with such stakeholders on actual progress (from interviews conducted in 2021); and to gain insight into key challenges and factors that have facilitated or hindered effective implementation to date.

## Method

### Design

This was a qualitative study. It analysed and compared interviews with senior stakeholders involved in primary care policy at national level in 2016 and 2021.

### Data

Data included in the analysis comprised a total of 12 transcripts from semi-structured interviews (six conducted in 2016; six in 2021) with key national stakeholders in primary care drawn from the main national organisations involved in primary care in Scotland. These organisations cannot be named for reasons of confidentiality as this might lead to identification of the stakeholders. Different stakeholders (from the same organisations) were interviewed in 2016 and 2021. Interviews were conducted either in person or via telephone, recorded, and transcribed verbatim, with the exception of two who did not wish to be recorded (in which case extensive notes were taken at the time of interview with their permission). All transcripts and interviewers’ notes were imported into NVivo (version 12 Pro), which was used to manage data organisation and analysis.

### Analysis

This study utilised a thematic framework approach to qualitative analysis of interview data.^8^ The efficacy of the framework method for use in medical and healthcare research is well supported.^9^ In the first phase of study, analysis of the 2016 interviews aimed to synthesise ideas around the expected impact of GP clusters and implementation challenges, as seen by senior Scottish primary care stakeholders in 2016. Interviews were coded inductively to generate a coding frame,^8,9^ which was continually refined as data analysis continued.^8,9^ Code summaries were written to allow for clustering of mutually compatible codes into initial categories. Abstraction of data were initiated through rigorous comparison and exploration of relationships among codes, drawing on the constant comparative method.^10^ As specific patterns emerged, all analysed interviews were re-examined to verify the presence or absence of these patterns. The completed framework facilitated the delineation of candidate themes, which acted as baseline ‘sensitising concepts’ with which to compare the ideas emerging from the 2021 stakeholder interviews.

During the second phase of study, the 2021 interviews were coded deductively using a framework derived from these sensitising concepts, while allowing for adjustment in light of the emergence of novel insights not captured by the predictions of the 2016 data. This phase of analysis provided insight into the stakeholders’ perceptions of the actual impacts and progress of the clusters since their introduction. Comparison of the 2016 stakeholder predictions with the 2021 stakeholder perceptions allowed conclusions to be drawn regarding the degree to which the expected barriers and facilitators to GP cluster working, and the predicted consequences of this quality improvement approach, have been actualised thus far.

The results section gives examples of quotes relating to the findings, but a fuller set of quotes are included in the Supplementary file 2.

## Results

### 2016: Predicted challenges and barriers

Overall in 2016, sufficient investment from the Scottish Government to provide adequate support to clusters was seen as central to ensuring the success of both the intrinsic and extrinsic roles. In particular, the requirement for data analytic support, administrative support, training in quality improvement, and project support were highlighted (see Supplementary file 2, Table S2.1). The impact of cluster working (particularly in relation to their extrinsic function) was predicted to be highly contingent on local relationships and engagement between clusters and Health and Social Care Partnerships (HSCPs), with recognition that cultivating this collaborative way of working could require a significant period of time (Supplementary file 2, Table S2.2). On a national level, facilitation to provide opportunities for clusters to come together to *'share understanding, share learning, excite people, show them the potential'* (Participant [P]3) was felt to be key (Supplementary file 2, Table S2.3).

Several factors were predicted relatively unanimously among stakeholders in 2016 as potential threats to effective cluster implementation. Deficits in motivation for engagement and understanding of the philosophy of cluster working among GPs working within the clusters (as well as members of the wider practice MDT [Supplementary file 2, Table S2.4]) emerged as one such factor. These views, based largely on the acknowledgement that the adoption of cluster working would require a significant *'culture shift'* (P5) are illustrated by quotes below.

One stakeholder expressed concern over the potential for scepticism among the profession in response to introduction of clusters on a wider scale, with the potential to negatively affect quality improvement activity:

*'I think the first barrier will be still lack of understanding of what it’s all about. There might be lack of belief or cynicism, "we’ve done all this before, it won’t work". There might be the "we can’t do this, we need you to tell us" … and if they are so inclined the barriers will stop them very easily from doing it.'* (P3)

Another suggested that a degree of 'resistance' was almost inevitable:

*'... from something new being introduced … doctors and other healthcare professionals who might be a bit reluctant to fully engage with something until they realise what it is they’re getting themselves into.' *(P9)

A similar view was expressed in a separate interview:

*'... opposition to change … realistically that’s what we’re going to find when we go and we try new ways of working … I think it was very much a mixed bag of ... of em opinion about clusters and whether that was the correct way to go forward.'* (P8)

There was speculation from one participant that this inertia may be more prominent among GPs nearing retirement:

*'... towards the end of their career, who will not wish to immerse themselves in new ideas when contemplating retirement.'* (P5)

Beyond this, an anticipated inadequacy of useful data was also of central concern. There was particular reference to the lack of national systems or ‘dashboards’ for data extraction (see Supplementary file 2, Table S2.5). Overcoming this issue was seen as fundamental to both the extrinsic and intrinsic functioning of clusters by providing the capacity to engage meaningfully with quality assurance, improvement, and planning based on robust assessment of local population health needs.

Additional key anticipated barriers related to perceptions of two important tensions inherent to the cluster model. These were as follows:

- a tension between intrinsic and extrinsic clusters roles: stakeholders questioned whether fulfilment of both roles would be mutually achievable in light of limited resources, and suspected that the intrinsic role may predominate to the detriment of the extrinsic role; and
- a tension between the aim of local autonomy and the need for a degree of national oversight: to ensure the element of governance and accountability required to safeguard consistent delivery of quality improvement across the country, while avoiding the *'micromanagement from the centre'* (P3) instilled by the QOF (Supplementary file 2.6).

### 2021: Actual challenges and barriers

**Figure 1.**
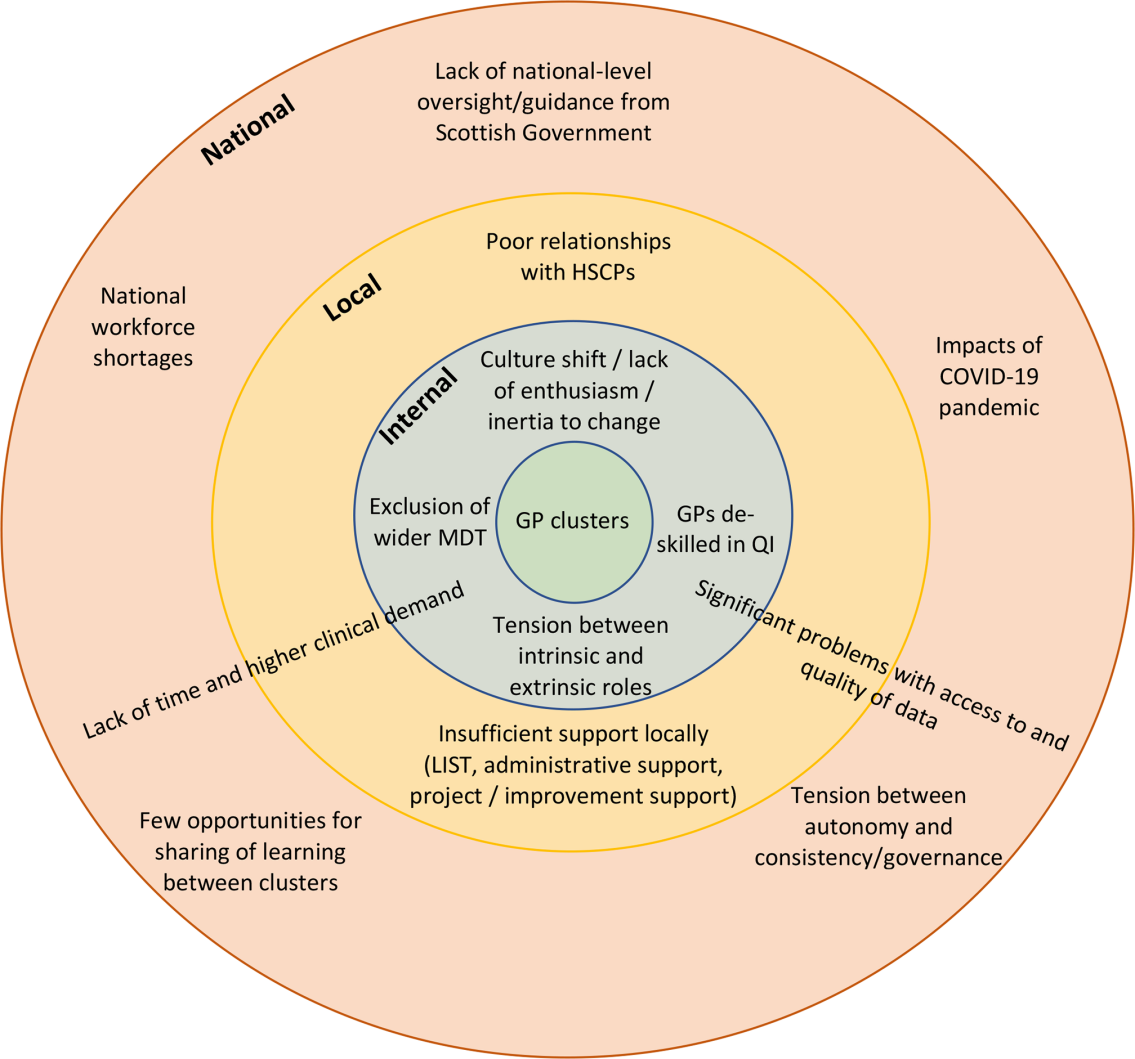
Internal, local, and national barriers to cluster success. HSCPs = Health and Social Care Partnerships. LIST = Local Intelligence Support Team. MDT = multidisciplinary team. QI = quality improvement.

In 2021, underdelivery of the very same provisions seen in 2016 to comprise the fundamental level of support required for clusters was held in part accountable for the perceived shortcomings of cluster efforts thus far. In addition, inadequate and inconsistent support in terms of funding of protected time for PQLs and CQLs was highlighted as a further limiting factor in 2021. This lack of appropriate facilitation was felt to be contributing to burnout among CQLs and PQLs, and disengagement from cluster roles. A summary of key identified barriers is shown in Figure 1.

Local relationships with HSCPs were seen as highly variable, based (as predicted) on pre-existing relationships within a locality, but remaining underdeveloped in many areas (Supplementary file 2 Table S2.2). Similarly, despite clear suggestions of intent in 2016 to provide opportunities for sharing of learning between clusters, the reported lack thereof emerged as one of the foremost concerns among stakeholders speaking in 2021 (Supplementary file Table 2.3). Interviews from 2021 suggested the actualisation of the major predicted barriers to cluster working noted in 2016 (Supplementary file 2, Table S2.5 and Table S2.6).

Additionally, three further issues, not clearly envisaged in 2016, emerged as key factors hindering the expected progress of clusters over the first 5 years of their implementation. First, many of the expectations placed on clusters by the Scottish Government and wider NHS were seen as largely unrealistic. In 2021, the lack of facilitation of clusters by the ‘wider system’ was juxtaposed with the unrealistic expectations that it placed on them.

The extrinsic role, in particular, was framed as *'one massive ask'* (Stakeholder [SH]01), which was *'very aspirational'*, as one stakeholder commented:

*'... you almost need a CQL with a diploma in public health to do that … one of the external roles is to reduce health inequalities … nobody else has managed to do that in 30 years … So it’s not a criticism, I just think, it’s very aspirational … with the amount of time they’ve got, I think it’s very difficult to do all that’s asked of them, that’s all.'* (SH01)

This idea of incompatibility between expectations and time provided to fulfil them was shared by another stakeholder:

*'... the time that clusters have to meet and undertake improvement is too small, it’s very small compared to the expectations around them.'* (SH02)

This stakeholder also highlighted additional demands in excess of those placed on clusters by the contract itself:

*'... expectations are placed on them from lots of different parts of the system, from primary care. But also from secondary care, you often hear, let’s ask the clusters to do it. Which of course was never the intention.'* (SH02)

Once again, this opinion was reiterated elsewhere:

*'I think everybody sees clusters as the answers to everything. And ask them to do hundreds of things that they can't possibly do.'* (SH01)

Second, national workforce shortages, relating crucially to GPs but also to the wider MDT, were seen as the central barrier limiting virtually all aspects of implementation of the new General Medical Services (GMS) contract. The resultant insufficient expansion of the primary care MDT, contributing to sustained pressure on PQLs and CQLs, was seen to be severely limiting capacity for engagement in cluster working, to the detriment of progress.

Expansion of the workforce was seen as the crucial missing piece on which converting *'the promise of the reforms'* (SH04) into practice was dependent. There was widespread recognition of the profoundly negative impact exerted by the primary care workforce crisis on the implementation of the new GMS contract and primary care reforms at large:

*'... the main problem is, there’s not enough staff.'* (SH01)

*'... while the principle is good, the reality is that there isn’t the workforce.'* (SH02)

*'... if we don’t have GPs the whole thing falls apart.'* (SH03)

Stakeholders who were involved directly in clinical work described the immense workload with which GPs currently faced, which they attributed to this lack of clinical capacity:

*'It comes back to the GP numbers again … I know there’s some days I’m going in that I’m doing the job of two people … When I have medical students … I mean I’m 56 years old — I often leave them ten metres behind me because I’m moving, physically moving so quickly … and that sort of pressure, the pressure of time that you’re working under.'* (SH03)

Despite allocation of protected time for CQLs and PQLs under the new contract, this was felt not to be working in practice owing to lack of clinical cover:

*'... we maybe said, okay, we’ll give you half a session a week, but there’s nobody to fill that surgery. There is no backfill so it’s work that’s done outwith those times.'* (SH05)

This time pressure was identified repeatedly as a barrier to cluster work, which limited CQL and PQL engagement:

*'... it’s very difficult then for our cluster leads to have the afternoon doing cluster work, when we've got patients queuing up, and not enough doctors to speak to them*.*'* (SH01)

*'... if you’ve got 20 patients waiting to see you and you’ve got a report on things like quality improvement in your cluster to read ... you’re going to see the patients.'* (SH03)

*'I think the practice quality leads are just too pushed.'* (SH01)

It also contributed to burnout:

*'... their work has probably felt quite frantic to them. I know some CQLs who have left, because they were just constantly working way beyond their hours ... '* (SH01)

And hindered achievement:

*'... we’ve got too much on our plate just now to do* [the clusters] *justice.'* (SH03)

*'... you can't get the existing workforce to do more than it’s doing now. So if you want the cluster leads to do more, which I think we need, then you know, we still need other GPs to do the work they're not doing.'* (SH01)

It was acknowledged that the work and time commitment required for meaningful engagement in clusters was substantial:

*'*[Cluster quality improvement work] *is a full-time job in many ways, to be able to do that well.'* (SH02)

In addition, the busyness of GP practices as a whole was raised as an important factor limiting the implementation of intrinsic, cluster-led quality improvement:

*'... if you’ve got a practice that is run off its feet, it may have a brilliant PQL who has protected time, but then you need MDT time … for that practice to actually* [do] *learning and development work in the practice.'* (SH01)

Lastly, the inevitably profound impact of the COVID-19 pandemic on clusters, and on general practice as a whole, could not be overlooked. Notably, however, stakeholders warned against the risk of overattributing the lack of progress among clusters to the pandemic, when in fact many problems identified predated its emergence in the UK. Further, several stakeholders highlighted some unexpected *'silver linings of COVID'* (SH04) that could potentially be taken forward by clusters.

## Discussion

### Summary

Predicted challenges in 2016 included balancing intrinsic and extrinsic roles, providing sufficient support, maintaining motivation and direction, and avoiding variation between clusters. Progress of clusters in 2021 was perceived as suboptimal, and was reported to vary significantly across the country, reflecting differences in local infrastructure. Practical facilitation (data, administrative support, training, project improvement support, and funded time) and strategic guidance from the Scottish Government was felt to be lacking. GP engagement with clusters was felt to be hindered by the significant time and workforce pressures facing primary care.

The end result could be viewed as a vicious cycle of non-progression whereby clusters become frustrated and burnout in the face of multiple system barriers impeding their work, leading to disengagement and further stagnation. Considerable investment in addressing these limiting factors will thus be required if clusters are to begin to exert a more meaningful impact on the NHS improvement landscape in Scotland.

### Strengths and limitations

The use of secondary analysis in this study enabled an in-depth analysis of themes relating specifically to GP clusters, using a subset of data from the parent study. However, critics have asserted that secondary analysis may be lacking in methodological rigour.^11^ Measures taken to ensure the credibility of the current analysis in light of such concerns included an emphasis on thorough data familiarisation and use of introspection (through personal reflection on positionality as a medical student and any related preconceptions or biases), as well as intersubjective reflection (through regular discussion of findings and emerging concepts or themes with supervisors) to ensure reflexivity in interpretation.^12,13^

Potential limitations to reaching data saturation relate to small sample size and exclusion of cluster members (CQLs and PQLs) as participants, both of which may potentially serve as sources of sampling bias within the data. Different stakeholders were interviewed in 2016 and 2021 from all the major national organisations involved in primary care. This provided an opportunity for findings to be influenced by individual differences between the groups, but simultaneously increased the breadth of stakeholder perspectives encompassed by the overall dataset for analysis. However, because only the views of the senior stakeholders have been reported, they cannot be compared with the views of other stakeholders, such as frontline GPs and members of the primary care MDT or patients. However, ongoing work is focusing on the views of these important groups.

### Comparison with existing literature

Findings from the present study are consistent with those from previous national^5–7^^,^^14^ and regional^15^ evaluations of Scottish GP clusters. Consonant with the current analysis, an observed failing in provision of a comprehensive support infrastructure is recapitulated across the literature as a critical factor limiting the impact of cluster working.^16^

It is notable that the relatively auspicious views of the early Inverclyde clusters, which emerged among interviews included in the present study, are somewhat starkly contrasted with findings from an in-depth evaluation of this project that highlighted multiple challenges faced by the Inverclyde clusters.^17^ This suggests that many apparently unforeseen barriers to cluster working were in fact evident within certain forums at an earlier stage.

When implementing healthcare policy change, drawing on the experiences of other nations has the potential to accelerate learning and inform strategic direction.^18^ Clusters have been operational in Wales since 2014 (although several important differences exist between the Welsh and Scottish models).^19^ Observations from a recent study involving Welsh cluster leads^20^ highlight many of the same challenges as were perceived among Scottish clusters and several suggestions for improvement that may be relevant to the situation in Scotland (for example, expansion of GP workforce, increased governance, and introduction of a national best-practice sharing programme). Further, studies on quality circles in Europe have elucidated key facilitators of success among these groups, including favourable social dynamics, group facilitation, training, access to data, protected time, and financial resources.^16,21,22^

### Implications for practice

Findings from the present study demonstrate that, 6 years into their period of operation, much work remains to be done to ensure ambitions for GP clusters in Scotland are realised. Importantly, enthusiasm for the ‘promise’ of the cluster model in contributing to the transformation of Scottish primary care has not been lost, despite frustration over slow progress. While the recent COVID-19 pandemic has undoubtedly impeded the progress of clusters, emergence from this situation engenders a unique context characterised by enormous flux in models of delivering primary care. Herein lies an opportunity for renewed commitment to ensuring the provision of the appropriate context and supporting infrastructure necessary for actualisation of this vision for GP clusters in Scotland. This study provides some contribution towards the more complete understanding of the range of barriers, as well as key facilitators, to effective cluster working that will be needed to inform the implementation of measures to accelerate the future development of GP clusters, and to support their meaningful contribution to improving the quality of primary care in Scotland.

It is noteworthy that, apart from the COVID-19 pandemic, most of the challenges reported by stakeholders in 2021 were predicted in 2016. It is imperative that these barriers, now surfaced in this and related work, are not ignored by policymakers and managers but acted on constructively with GPs to rectify the situation and prevent further loss of morale and GP burnout.
